# Does Ramadan Fasting Adversely Affect Cognitive Function in Young Females?

**DOI:** 10.1155/2015/432428

**Published:** 2015-11-30

**Authors:** Mahboubeh Ghayour Najafabadi, Laya Rahbar Nikoukar, Amir Memari, Hamed Ekhtiari, Sara Beygi

**Affiliations:** ^1^Neuroscience Institute, Sports Medicine Research Center, Tehran University of Medical Sciences, Tehran, Iran; ^2^Faculty of Sports Sciences and Physical Education, University of Tehran, Tehran, Iran; ^3^Research Center for Molecular and Cellular Imaging, Tehran University of Medical Sciences, Tehran, Iran; ^4^Translational Neuroscience Program, Institute for Cognitive Science Studies, Tehran, Iran

## Abstract

We examined the effects of Ramadan fasting on cognitive function in 17 female athletes. Data were obtained from participants of two fasting (*n* = 9) and nonfasting (*n* = 8) groups at three periods of the study (before Ramadan, at the third week in Ramadan, and after Ramadan). Digit span test (DST) and Stroop color test were employed to assess short-term memory and inhibition/cognitive flexibility at each time point. There were no significant changes for DST and Stroop task 1 in both groups, whereas Stroop task 2 and task 3 showed significant improvements in Ramadan condition (*p* < 0.05). Interference indices did not change significantly across the study except in post-Ramadan period of fasting group (*p* < 0.05). Group × week interaction was significant only for error numbers (*p* < 0.05). Athletes in nonfasting showed a significant decrease in number of errors in Ramadan compared to baseline (*p* < 0.05). The results suggest that Ramadan fasting may not adversely affect cognitive function in female athletes.

## 1. Introduction

All around the world millions of Muslims observe the religious ritual of fasting during the holy month of Ramadan every year. They are required to abstain from eating, drinking, and sexual contacts from sunrise to sunset. However there are some exceptions to the rules; for instance, women could break the Ramadan fasting during the menstruation period. The duration of each episode of fasting varies depending upon the solar calendar dates and geographical locations it occurs in. In summer months it may last more than 16 hours a day. Healthy and sedentary individuals have been frequently investigated through Ramadan fasting [1, 2]. Recently, sportspeople have also received the attention of researchers in the case of Ramadan and physical performance [3–5]. Previous data showed that the effect of Ramadan on athletic cognitive or physical performances is mixed [6].

Interestingly experimental fasting studies also showed mixed findings; limited research suggested that short-term caloric deprivation adversely affects cognition though other studies revealed that cognitive performance and activity are not adversely affected by caloric deprivation [7, 8]. These studies often examined the effects of near-total caloric deprivation on individual cognitive or physical function in a controlled environment.

Dehydration is another factor shown to have detrimental effects on cognitive function through several studies [9, 10]. However, most of them employed heat stress, exercise, or a combination of both to induce dehydration and only few studies have used voluntary fluid deprivation as their main methodology [11–13]. Although one study indicated a mental deterioration during 24 h voluntary fluid deprivation [11], another showed that 24 h water deprivation with 2.6% weight loss did not alter the results of psychomotor assessments including choice reaction time task, manual tracking task, and the Stroop color test [13]. Nevertheless, the results due to experimental fasting cannot be extrapolated simply to Ramadan fasting, since unique features of this practice such as intermittent type and environmental changes have not been included. Therefore it is required to investigate Ramadan-specific variables which might be associated with mental performance [14, 15].

Eating exclusively at nighttime imposes significant alterations on individuals' life style and sleep-wake cycles; therefore food and fluid deprivation theory does not appear to be a convincing explanation for all the presumed impacts of Ramadan fasting. The studies described that combination of changes in sleep-wake cycle, food and fluid intakes, and circadian rhythms was likely to affect mental, physical, and social performances [16]. Several subjective or objective assessments on the sleep patterns during Ramadan fasting reported sleep architecture alterations, delayed bedtime, and a significant reduction in nocturnal sleep [17, 18].

Their findings showed that sleep loss might be responsible for excessive fatigue and reduced alertness in the daytime. Ramadan fasting also imposes deep impacts on the natural circadian rhythm through significant shifts in sleep patterns, body clock, and other physiological indices [16, 19]. Irritability could be increased during Ramadan fasting which was attributed to reduction in sleep time or nicotine withdrawal [20]. Similarly, impairments in vigilance, memory, and continuous attention have been suggested in the course of Ramadan [21]. Increased reaction time has also been noted at the beginning of Ramadan fasting [22].

Although physiologic indices and physical performance changes imposed by Ramadan fasting in athletes had been a field of interest in the past few years [23, 24], little information is still available on the athletes cognitive alterations during Ramadan fasting. In a recent study on male athletes, Ghanouni et al. [25] found slight increase in recognition reaction time at the beginning of Ramadan which progressively improved across the remainder of fasting period, whereas the critical flicker fusion and motor reaction time were not affected. The research to date has tended to focus on males rather than females and no research has investigated cognitive variations in female athletes during Ramadan. The objectives of this study are to determine whether Ramadan fasting imposes adverse effects on cognitive function in elite female athletes.

## 2. Methods

### 2.1. Participants

Seventeen volunteer elite female sprinters aged 13–24 years were recruited for the study. They came from a club of track and field located in the middle zone of Tehran. Nine of the participants who voluntarily chose to observe Ramadan fasting comprised fasting group (FG) and eight of them were voluntarily assigned to nonfasting group (NFG). The participants were controlled for menstrual-cycle status particularly for testing days. They were all enrolled in the study after receiving adequate verbal explanations about the research procedures and providing the informed consents. The protocol was approved by the Ethic Committee of Teheran University of Medical Science.

### 2.2. Procedure

The prospective two-cohort study was conducted during the Ramadan fasting month. Participants were examined at three different points: two weeks before Ramadan, 3rd week of fasting, and two weeks after the end of Ramadan. Participants were examined only if they were not in their menstrual period. All participants were examined at a fixed time, between 4 and 5 p.m., in a private setting at the track gym. It was just before the beginning of their usual training sessions which was about two hours before Iftar (the first meal taken after sunset). All the players were adhered to a weekly regular training program before and during Ramadan. Cognitive function was evaluated as memory and cognitive flexibility.

### 2.3. Measures

#### 2.3.1. Digit Span Test (DST)

Typical verbal digit span test was used to measure short-term memory. This test is a component of the Wechsler Adult Intelligence Scale, Fourth Edition [26], and reflects a part of the verbal IQ. The test consisted of a direct and a reverse subtask. The direct subtask began with three numbers, increasing until the examinee was unable to repeat the sequence correctly. The numbers were read aloud at a rate of one per second and at the end of each sequence participants were asked to repeat the numbers in the same order. If they failed two consecutive sequences with the same length the direct subtask was stopped. Then the reverse subtask was performed in the same manner except that it began with two numbers and had a maximum of eight and participants were supposed to repeat the numbers in the reverse order. Acquired score in each subtask was the number of correctly repeated strings, with two strings being presented for each length. The final score was calculated by the sum of two scores acquired in direct and reverse subtests.

#### 2.3.2. Stroop Color Test

Paper Stroop color test was employed to assess inhibition and cognitive flexibility [27]. It consisted of three subtasks taking 45 seconds in each one. In the first subtask four basic color words including red, blue, yellow, and green printed in black ink were presented as 100 items per page in a random order. Subtask 2 displayed solid color squares and subtask 3 provided the participants with color words printed in an incongruent ink color (e.g., the word “blue” printed in red ink). In subtask 1 participants were required to read as more words as they could. In subtask 2 they were supposed to name the colors and in the 3rd subtask they were instructed to name the ink colors regardless of the color words. The number of correct items mentioned by each participant was recorded at three different stages of the test as word score, color score, and color-word score, respectively. Finally an interference index was calculated by subtracting the number acquired in the word-color incongruent subtask from the number recorded in the color subtask suggested by Golden and Freshwater [27]. The number of errors which occurred within the total duration of a single Stroop test was also recorded for each participant [28].

### 2.4. Statistical Analysis

The data were analyzed using two-factor repeated-measures analysis of variance (*ANOVA*) with one repeated factor (weeks) and one grouping factor (fasting versus nonfasting). Post hoc analysis with Bonferroni correction was also employed where appropriate. The *p* values less than 0.05 were considered the level of significance. Statistical analysis was performed by Statistical Package for the Social Sciences (*SPSS*), version 17.

## 3. Results

Nine female athletes aged 17.6 ± 3.6 years in FG and eight female athletes aged 20.6 ± 2.7 years in NFG were observed.

### 3.1. DST

**Figure 1:**
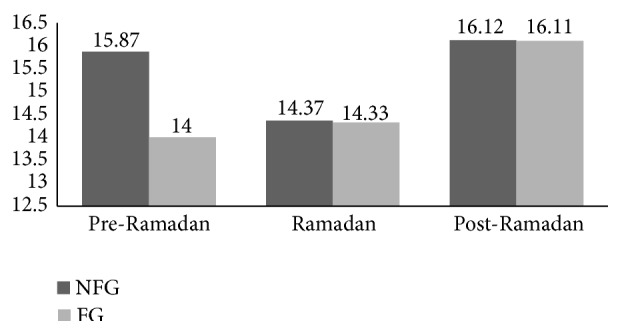
Digit span test (DST) scores at three occasions in both fasting group (FG) and nonfasting group (NFG).

There was no main effect of fasting on DST scores, although there was an improvement in NFG post-Ramadan values compared to Ramadan (*p* = 0.006, 95% CIs [0.605, 2.895]). Similar significant increase was detected in FG records of the post-Ramadan period compared to pre-Ramadan period (*p* = 0.03, 95% CIs [0.201, 4.021]). There was also no significant interaction between weeks and groups (see Figure 1).

### 3.2. Stroop Color Test

Statistical analysis of word scores revealed no significant main effect of time and also no significant interaction between weeks and groups of athletes (see Table 1). There was a main effect of week for color scores in FG (*p* = 0.047) and NFG (*p* = 0.03). Players showed an improvement in Ramadan compared to pre-Ramadan period in both FG (*p* = 0.041, 95% CIs [0.189, 8.478]) and NFG (*p* = 0.037, 95% CIs [0.493, 15.007]). There were no between-group differences for color scores in three different testing occasions. There was not also a significant interaction between groups and weeks (see Table 1).

There was a main effect of week for color-word scores in both groups (*p* < 0.001). Significant improvements were observed in athletes performance in Stroop subtask 3 on Ramadan compared to pre-Ramadan occasion in either FG (*p* < 0.001, 95% CIs [2.976, 7.912]) or NFG (*p* = 0.019 with 95% CIs [1.543, 14.957]). The increasing trend was shown to be continued to post-Ramadan state only in fasting players (*p* = 0.024). As shown in Table 1, between-group difference for color-word scores was only found in Ramadan (*p* = 0.04) but not in pre- or post-Ramadan occasion. Similar to other variables, no significant interaction was found between groups and weeks.

There was a main effect of week for interference indices in FG (*p* = 0.003). On the Ramadan occasion no significant changes were found for both groups, compared with baseline values. Only FG participants showed a significant improvement on post-Ramadan occasion (*p* = 0.038, 95% CIs [0.472, 15.973]). There were no between-group differences for interference indices in three different testing occasions. No significant interaction was detected between groups and weeks for interference indices (see Table 1).

There was a main effect of week for the number of errors in NFG (*p* = 0.003) unlike the FG. Athletes in NFG demonstrated a significant decrease in number of errors in Ramadan compared to baseline (*p* = 0.018, 95% CIs [0.623, 5.877]). For this variable, a significant interaction was identified between groups and weeks (*p* = 0.037) (see Table 1). Adjustment for age did not reveal new results for DST and Stroop variables as well.

## 4. Discussion

Although various studies aimed to explore different aspects of Ramadan fasting, the extent of works on athletes has been limited solely to examining their physical performance, body composition, and recently metabolic and biochemical alterations [18, 29, 30]. However, there have been no controlled studies which investigate cognitive function during Ramadan and this is the first time that impact of Ramadan fasting on cognitive performances was examined in female athletes. The most interesting finding was that fasting imposed no adverse effects on short-term memory and cognitive flexibility function in female athletes.

Given to distinctions between male and female mental skills [31, 32], female cognitive system might differently respond to Ramadan fasting. Indeed the findings from male studies could not be generalized to female athletes. For the first time we documented such findings in female athletes. On the post-Ramadan occasion, fasting group showed an improvement in interference indices from Stroop task; however women in nonfasting group showed no significant changes in interference indices. Although it could be a result of increased familiarity with the test procedures, the unfavorable effects of fasting which were eliminated in the recovery condition may also be suspected. Another important finding was the decrease of error numbers in Ramadan in nonfasting but not fasting athletes. Since fasting participants showed no improvement in error scores, it may hypothesize that error proneness was adversely affected by Ramadan fasting. However both FG and NFG did not show significant alterations in performance on the word subtask of Stroop test throughout the study occasions. Indeed with regard to the simplicity of word reading, it provides a context to interpret the results regardless of training effect.

Ramadan fasting may not adversely influence women at least in some cognitive functions. The current results which are consistent with previous studies showed stable cognitive performance across experimental calorie restriction [8, 33]. Researchers attributed intact memory function to stable glucose levels, since deterioration of cognitive performance has been previously observed with blood glucose levels less than 3.6 mmol^−1^ in either normal or clinical subjects [34, 35]. Several Ramadan studies also reported that serum glucose concentrations will remain stable during the fasting period in either athletic or nonathletic population [36–38]. It had been assumed that the glycogen resources were supported by the large meals taken during nighttime of Ramadan but also the compensatory gluconeogenesis mechanisms [39]. Therefore the preserved memory function in the fasting condition may be partly related to tight endogenous regulation of blood glucose levels.

On the other side our findings are in agreement with Szinnai et al. [13] study on voluntary fluid deprivation. They found no significant changes in interference indices after 24 h water deprivation and suggested that the healthy individuals are able to adapt to slowly induced water deficits. They also reported superior mental effort for task completion during dehydration compared with control phase. Therefore it could be hypothesized that young healthy individuals would compensate for possible adverse effects of fluid deprivation during Ramadan by upgrading their mental effort and motivation [40, 41]. It could also serve as a good explanation for unchanged objective performance in psychomotor tasks despite the worsened subjective feelings during Ramadan fasting reported by Roky et al. [22]. Furthermore some studies addressed a deterioration of psychomotor performance at the beginning of Ramadan which was progressively reversed across the fasting period [22, 25]. A similar pattern was also reported for subjective feeling variations during Ramadan [42]. Similar findings on memory and Stroop test were also reported from fasting studies in other religions (i.e., Jewish fasting) [43]. These findings suggest that a neuropsychological adaptation might be developed in the context of intermittent fasting. However, further biobehavioral investigation is warranted to validate this hypothesis.

However our results differed from some published studies [21, 44] which indicated cognitive deterioration during Ramadan fasting. A possible explanation for this is due to the evident differences in cognitive profile of athletic and nonathletic population; it has been frequently indicated that sport activities were associated with higher performance in various cognitive domains [45, 46]. In addition to relatively higher cognitive performance in athletes, our study implies that they may be also less susceptible to fasting induced cognitive impairments. However, it needs to be more elaborated and confirmed through further investigations in the future.

The results of the present study should be interpreted in the context of its limitations such as the small sample sizes of each group which might result in limited statistical power to discriminate between groups and occasions. Another limitation is due to status of menstrual cycle in participants of this study. Since cognitive performance could be modulated by the menstruation cycle, we could not explain whether menstrual cycle had an effect on females' cognitive function or not. Interestingly based on a recent review study [47], there is no sufficient evidence to support hypotheses that sex dimorphic cognitive skills are improved or aggravated during menstrual cycle phases with low or high estrogen. However few studies suggested improved memory performance at times of high estradiol levels. Sleep deprivation or disruption could not be ruled out as possible confounders in this study. However to minimize this effect, we instructed all participants to complete their sleep time on their regular basis (i.e., nonfasting periods). Furthermore, it seemed better to employ a comprehensive battery of tests measuring a wide variety of cognitive domains in order to maximize the sensitivity of assessments. It is also recommended that future studies may investigate the daytime effect on cognitive variables in Ramadan curriculum. Finally although this study primarily aimed to assess cognitive performance, future studies should examine sport performance in addition to cognitive performance of female athletes.

## 5. Conclusion

By keeping in mind the preliminary findings discussed above it is shown that Ramadan fasting may not have adverse effects on specific cognitive performances in female athletes.

## Figures and Tables

**Table 1:** Stroop test scores at three occasions in fasting group (FG) and nonfasting group (NFG) (mean values with standard deviations in parentheses).

Variable	Group	Pre-Ramadan	Ramadan	Post-Ramadan	*p* values for week × group interaction
Word score	NFG	123.9 (10.1)	125.6 (19.7)	126 (15.7)	0.6
	FG	108.2 (10.4)	113.8 (7.7)	113.3 (8.3)	

Color score	NFG	76.5 (9.7)	84.3 (14.5)^*∗*^	84.4 (9)^*∗*^	0.3
	FG	75.3 (7.2)	79.7 (6.4)^*∗*^	78.2 (6.4)	

Color-word score	NFG	44.8 (8.3)	53 (7.7)^*∗*^	55.8 (4.7)^*∗*^	0.2
	FG	42.4 (7.2)	47.9 (6.9)^*∗*^	54.7 (6)^*∗∗*^	

Interference index	NFG	31.8 (7.5)	31.3 (8.5)	28.6 (8)	0.2
	FG	32.9 (9.2)	31.8 (7.1)	23.6 (5.8)^*∗∗∗*^	

Error number	NFG	4.3 (2.2)	1 (0.8)^*∗*^	2 (1.2)	0.03
	FG	2.7 (1.7)	1.9 (0.8)	2.2 (1.1)	

*Note*. ^*∗*^Significantly different from pre-Ramadan condition (*p* < 0.05).

^*∗∗*^Significantly different from Ramadan condition (*p* < 0.05).

^*∗∗∗*^Significantly different from pre-Ramadan and Ramadan conditions (*p* < 0.05).
